# Glatiramer Acetate Treatment Normalizes Deregulated microRNA Expression in Relapsing Remitting Multiple Sclerosis

**DOI:** 10.1371/journal.pone.0024604

**Published:** 2011-09-16

**Authors:** Anne Waschbisch, Monika Atiya, Ralf A. Linker, Sergej Potapov, Stefan Schwab, Tobias Derfuss

**Affiliations:** 1 Department of Neurology, Friedrich-Alexander University, Erlangen-Nürnberg, Germany; 2 Institute of Medical Informatics, Biometry and Epidemiology, Friedrich-Alexander University, Erlangen-Nürnberg, Germany; 3 Department of Neurology, University Hospital Basel, Basel, Switzerland; Julius-Maximilians-Universität Würzburg, Germany

## Abstract

The expression of selected microRNAs (miRNAs) known to be involved in the regulation of immune responses was analyzed in 74 patients with relapsing remitting multiple sclerosis (RRMS) and 32 healthy controls. Four miRNAs (miR-326, miR-155, miR-146a, miR-142-3p) were aberrantly expressed in peripheral blood mononuclear cells from RRMS patients compared to controls. Although expression of these selected miRNAs did not differ between treatment-naïve (n = 36) and interferon-beta treated RRMS patients (n = 18), expression of miR-146a and miR-142-3p was significantly lower in glatiramer acetate (GA) treated RRMS patients (n = 20) suggesting that GA, at least in part, restores the expression of deregulated miRNAs in MS.

## Introduction

microRNAs (miRNAs) have recently emerged as potential biomarkers of disease in different autoimmune disorders including rheumatoid arthritis [1], systemic lupus erythematosus [2] or Sjögren's syndrome [3]. miRNAs are short (∼22 nucleotides) non-coding RNA molecules that regulate gene expression at the post-transcriptional level. One miRNA may target several hundreds of messenger RNAs and it has been proposed that up to 50% of mammalian genes underlay miRNA fine-tuning [4]. miRNAs are critically involved in the regulation of a wide array of cellular and developmental processes including the differentiation of immune cells and the outcome of immune responses [5].

Recent data indicate that miRNA dysregulation may also contribute to the pathogenesis of multiple sclerosis (MS): Overexpression of miRNAs that target CD47 was found to contribute to macrophage-mediated damage in active MS lesions and miRNA expression profiling in whole blood or leukocytes derived from MS patients suggests an aberrant expression of various miRNAs in the peripheral immune compartment [6], [7]. Here we studied the expression of five selected immunologically relevant miRNAs in peripheral blood mononuclear cells (PBMC) derived from patients with relapsing-remitting multiple sclerosis (RRMS) and healthy controls and addressed the impact of immunomodulatory therapy on miRNA expression by comparing treatment-naïve to glatiramer acetate (GA) or interferon-beta (IFN-beta) treated patients.

## Results

### Overexpression of miR-142-3p, miR-155, miR-146a and miR-326 in PBMC of RRMS Patients

**Figure 1:**
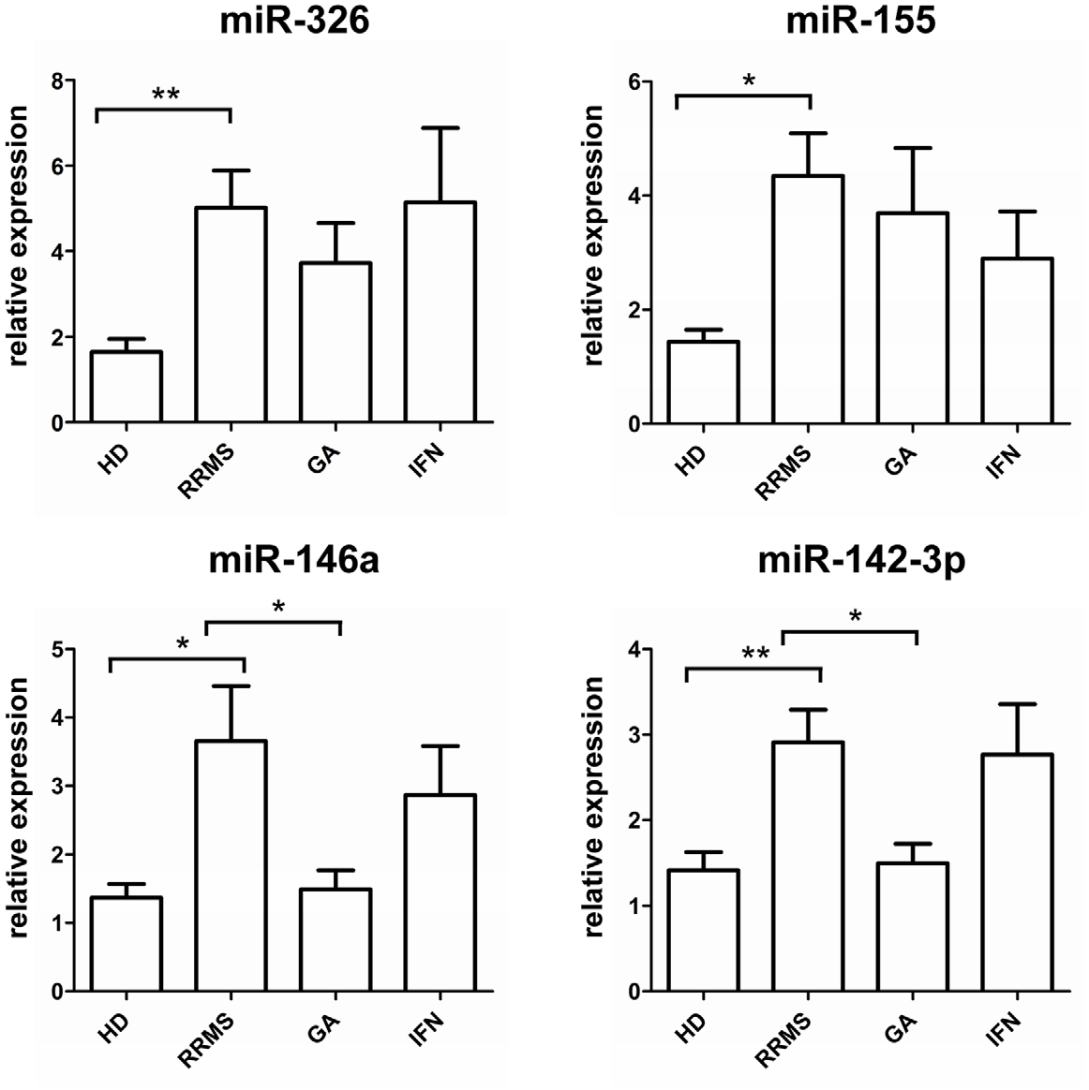
miRNA dysregulation in MS and the impact of immunomodulatory treatment. The relative expression of the mature miRNAs miR-326, miR-155, miR-146a and miR-142-3p in PBMC derived from healthy donors (HD, n = 32), treatment-naïve patients with RRMS (n = 36) and glatiramer acetate (GA, n = 20) or Interferon-beta (IFN, n = 18) was analyzed by real-time PCR. Bars represent the mean + S.E.M. * p<0,05; **p<0,005.

**Figure 2:**
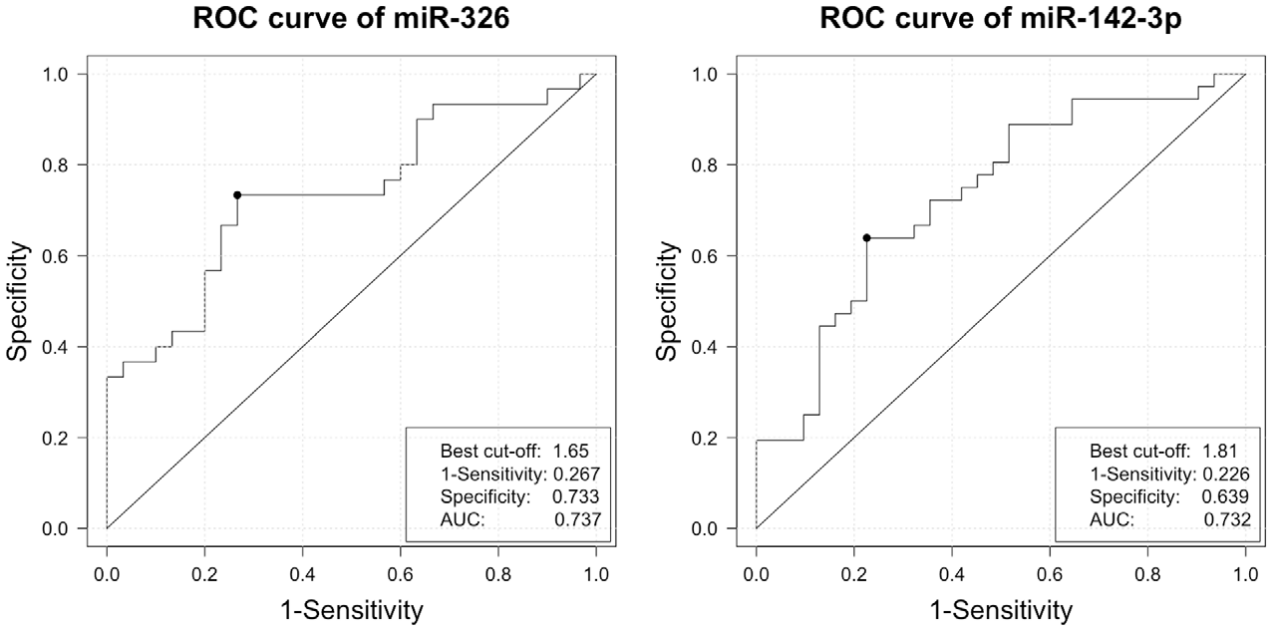
ROC analysis. To assess the sensitivity and specificity of miRNAs to predict disease, receiver operator characteristic curves were computed based on the relative miRNA expression in treatment-naïve patients and healthy controls. The ROC curves for miR-326 and miR-142-3p are exemplary depicted.

We studied the expression of five selected miRNA (miR-20b, miR-142-3p, miR-146a, miR-155 and miR-326) in PBMC derived from treatment naïve patients with RRMS (n = 36) and healthy controls (HC, n = 32). While no significant differences could be detected for the expression of miR20b, the expression of miRNAs miR-142-3p, miR-146a, miR-155 and miR-326 was remarkably increased (between 2 and 3 fold) in untreated RRMS patients compared to healthy controls (Figure 1). No significant correlations were found between the expression of miRNA and age or gender (data not shown). To analyze the diagnostic potential of individual miRNAs to predict disease, we computed receiver operator characteristic curves (ROC) for each miRNA based on the data from treatment-naïve patients and healthy donors. The discriminatory power of individual miRNA was low with an area under the curve ranging from 0.67- 0.74. The ROC curves of miR-326 and miR-142-3p are exemplary depicted (Figure 2). To investigate whether a combination of any of these miRNAs may be more accurate in predicting disease, a stepwise quadratic discriminant analysis was performed and evaluated using the leave-one-out cross-validation technique. The combination of miR-155, miR-146a and miR-142-3p yielded the best results with a sensitivity of 77.8% and a specificity of 88.0% in predicting disease (AUC 0.77).

### Aberrant Expression of miR-142-3p and miR-146a is Restored in GA Treated MS Patients

In parallel we analyzed the expression of miRNA in a group of glatiramer acetate (n = 20) and interferon-beta (n = 18) treated RRMS patients to detect a potential impact of immunomodulatory therapy on deregulated miRNA. The expression of miR-142-3p, miR-146a, miR-155, miR-326 and miR-20b did not differ between treatment-naïve and IFN-beta treated RRMS patients. In contrast, the expression of miR-142-3p and miR-146a was significantly decreased in GA treated RRMS patients to values that resembled the level of normal (p = 0,003; p = 0,028). miR-155 and miR-326 did not differ between untreated and the glatiramer acetate treated group (Figure 1). *In vitro* stimulation of PBMC derived from untreated MS patients with GA for up to 72 hours (40 µg/ml) did not result in the downregulation of the miRNAs under investigation suggesting that the effects of miRNA modulation by GA are of more complex nature requiring long-term GA exposure or interaction with resident cells of secondary lymphoid organs (Figure S1).

## Discussion

Emerging evidence supports a critical role of miRNAs in the pathogenesis of autoimmune diseases including multiple sclerosis [5], [6]. miRNA profiling studies in MS patients have repeatedly demonstrated an aberrant expression of specific miRNA in cells of the peripheral blood and deregulated miRNAs have been proposed as diagnostic biomarkers of the disease [8]-[13]. However, the results of these studies were contradictory which may be secondary to patient selection (disease activity [13], immunomodulatory treatment [11], inclusion of progressive MS [8], ethnic origin [10]), sampling of different materials (whole blood [8], [11], leukocytes [13], lymphocyte subsets[9], [10]) and differences in the chosen screening method (microarray analysis[8], [11], microfluidic TaqMan arrays [12], [13]). Accordingly, although several miRNAs have been proposed as biomarkers in different states of the disease, validation in a well-defined and homogenous population of patients with RRMS is still required. Therefore we decided to analyze the expression of selected, immune relevant miRNAs by quantitative PCR in a cohort of treatment-naive RRMS patients and healthy controls.

Two of the miRNAs found to be overexpressed in MS, miR-326 and miR-155 have recently been identified as crucial regulators of T cell development and Th17 differentiation [10], [14] and were found to promote CNS inflammation in experimental autoimmune encephalomyelitis (EAE). The first-time demonstration of miR-326 and miR-155 overexpression in PBMC derived from European MS patients underlines the clinical significance of these findings and adds to previous data on miR-326 dysregulation in Chinese patients with relapsing MS [10].

While the expression levels of miR-326 and miR-155 did not differ between untreated and GA or IFN-beta treated patients, GA treatment seemed to normalize miR-146a and miR-142-3p expression in MS patients. miR-142-3p expression has been linked to immune tolerance, since it was found to be repressed by FOXP3 resulting in an increased production of cyclic AMP and suppressor function in T regulatory cells [15]. Here we demonstrate upregulation of miR-142-3p in PBMC from RRMS patients which is in line with a previous report [11]. The mechanisms by which GA influences the expression of miR-142-3p remain unknown. However, it is well perceivable that an expansion of T regulatory cells by glatiramer acetate and/or changes in the composition of the T cell compartment may account for the downregulation of miR-142-3p.

miR-146a has emerged as a key player in the regulation of innate immunity and seems to be critical for the suppressor function of T regulatory cells [16]. miR-146a was found to be overexpressed at the site of inflammation in RA [17], psoriasis [18] and within active MS lesions [19]. We hypothesize that overexpression of miR-146a in PBMC of MS patients is reactive to the proinflammatory milieu in MS. In this light, downregulation of miR-146a in the glatiramer acetate treatment group may reflect restoration of the cytokine milieu by GA dependent induction of a Th1 to Th2 shift and inhibition of monocyte reactivity [20]–[22].

There are certain possible confounders to this study. The mean age and disease duration was higher in GA and IFN treated patients compared to treatment-naïve patients which is an obvious consequence of an early treatment strategy. Since miRNA expression in PBMC may change with age and immune senescence or may be influenced by a chronic inflammatory state we analysed whether expression of the studied miRNAs was biased by age or disease duration. However, we did not find significant correlations of the miRNA expression levels with age or disease duration in our study.

The patients we studied represent a rather active MS population as reflected by the high percentage of patients that experienced relapse within the last 3 months (up to 39% within the no treatment group). It has been suggested that certain miRNAs levels may increase during MS relapse [10], [13]. Since recent MRI as a surrogate marker of disease activity was not available in most of the patients we cannot control a possible influence of disease activity on miRNA expression in our study which may have biased our results.

Summarizing, we report the aberrant expression of four miRNAs, previously reported to be critically involved in TH17 differentiation (i.e. miR-326, miR-155), the regulation of immune tolerance (miR-142-3p, miR-146a) or innate immunity (miR-146a). Immunomodulatory treatment with IFN-beta did not restore the expression of deregulated miRNAs, whereas GA treatment seemed to normalize the levels of miR-146a and miR-142-3p but not miR-155 and miR-326 expression in RRMS patients. The biologic significance of our findings is further underlined by the fact that the overexpressed miRNA in peripheral blood leukocytes, were also among the most significantly upregulated miRNAs in active MS lesions in a seminal study by Junker et al. [19]. In conclusion, our study adds to the emerging evidence of miRNA dysregulation in MS.

## Materials and Methods

### Patients and Sample Collections

The study was approved by the ethics committee of the Friedrich-Alexander University Erlangen (No. 4203) and written informed consent was obtained from all participants. RRMS patients that had been diagnosed according to the McDonald criteria were eligible for participation in this study. Patients had to be either treatment naïve, or treated with the immunomodulatory drugs glatiramer acetate (GA, Copaxone®) or Interferon-beta (IFN-beta, Avonex® n = 5, Rebif® n = 8, Betaferon® n = 5) for at least 3 months. Patients that were treated with glucocorticoids during the last four weeks before study entry were excluded from the study. >97% of patients and healthy controls were of Caucasian origin. All patients were assessed for EDSS and clinical parameters of disease activity by their treating physician. Patients that had been relapse-free for at least 3 months were considered as clinically stable. Peripheral blood was obtained by venipuncture and immediately processed for isolation of PBMCs. Demographic and clinical details are summarized in table 1.

**Table 1:** Clinical and demographic details of patients and healthy controls.

	mean age +/-SD	f:m ratio	median EDSS	EDSS range	> 3 months relapse free	mean disease duration +/− SD
**Healthy Controls**	34.5 +/− 10.7 a	1.7∶1	n/a	n/a	n/a	n/a
**treatment-naïve RRMS**	36.6 +/− 9.6 a	2.0∶1	1.5	0-3.5	61%	5.1 +/− 6.1 a
**Glatiramer Acetate** † **treated RRMS**	40.5 +/− 12.3 a	1.9∶1	1.75	0-4.0	75%	9.4 +/− 7.6 a
**IFN-beta** ‡ **treated RRMS**	38.8 +/− 6.7 a	2.6∶1	2	0-5.5	83.3%	8.8 +/− 7.8a

† Copaxone®;

‡ Avonex® n = 5, Betaferon® n = 5, Rebif® 22 n = 4, Rebif® 44 n = 8; SD: standard deviation.

### Isolation of PBMCs and RNA Isolation

PBMCs were isolated from EDTA blood via ficoll density gradient centrifugation. 1×10^7^ cells were resuspended in Qiazol® (Qiagen, Hilden, Germany) and stored at −80°C. RNA enriched in miRNA was isolated using the miRNAeasy® Mini and RNeasy® MinElute® Cleanup Kit according to the manufacturer's protocol. Total RNA concentrations were determined using a NanoDrop spectrophotometer (NanoDrop technologies, USA).

### *In Vitro* GA Stimulation of PBMC

PBMC were cultured in RPMI 1640 (Gibco Invitrogen GmbH, Karlsruhe, Germany) supplemented with 10% of FCS (PAA, Pasching, Austria), glutamine and antibiotics. Cells were grown at a densitiy of 2×10^6^ ml in 6-well plates (Gibco Invitrogen GmbH, Karlsruhe, Germany) under standard culture conditions (37°C, 5%CO_2_). GA (Copaxone®, 40 µg/ml) was added to the medium for different periods of time. RNA was isolated as described above.

### Real-Time PCR

The TaqMan® MicroRNA Reverse Transcription Kit was used according to the manufacturer's instruction for reverse transcription with target specific stem loop primers provided in TaqMan® miRNA Assays. The TaqMan Universal MasterMix was used according to the manufacturer's protocol for amplification of the targets on an ABI PRISM® 7900 HT Real Time PCR System. The following TaqMan miRNA assays were used: hsa-miR-155, Assay ID 002623; hsa-miR-326, Assay ID 000542; hsa-miR-142-3p, Assay ID 000464; hsa-miR-146a, Assay ID 000468; has-miR-20b, Assay ID 001014. RNU6B (Assay ID 001093) that has been previously used in several studies analyzing miRNA expression in immune cells^10–12^ was used as an endogenous control to calculate ΔCT. All samples were analysed in triplicates. The average ΔCT of healthy donors was used as a calibrator to calculate ΔΔCT. Results are expressed as 2^-ΔΔCT^.

### Statistical Analysis

Differences in miRNA expression were determined using nonparametric tests (Mann-Whitney-U, Kruskal-Wallis-Test with Dunn's multiple comparison). p-values <0.05 were considered significant. Correlation of miRNA expression with age, disease duration and gender was assessed by calculating the spearman and point-biserial correlation coefficient. Receiver operator characteristic curves (ROC) were computed and the area under the curve (AUC) was calculated to assess the discriminatory power of individual miRNA. A quadratic discriminant analysis (QDA) was employed to assess the predictive power of a combination of miRNAs to differentiate between healthy controls or RRMS patients. The adapted model was evaluated by the leave-one-out cross-validation method. Statistical analysis was performed using the R-system for statistical computing [23] (version 2.12.2, R Development Core Team 2011) and GraphPad Prism (version 5.0, GraphPad Software Inc.).

## Supporting Information

Figure S1 **miRNA expression was analyzed in PBMC derived from RRMS patients (n = 6) after** ***in vitro*** **treatment with glatiramer acetate (GA, 40 **µ**g/ml) for the indicated periods of time.**
(TIF)
