# Supplementary figures and images for: Glatiramer Acetate Treatment Normalizes Deregulated microRNA Expression in Relapsing Remitting Multiple Sclerosis
Source: PLoS One. 2011 Sep 16;6(9):e24604. doi: 10.1371/journal.pone.0024604 (PMC3174971; doi:10.1371/journal.pone.0024604)

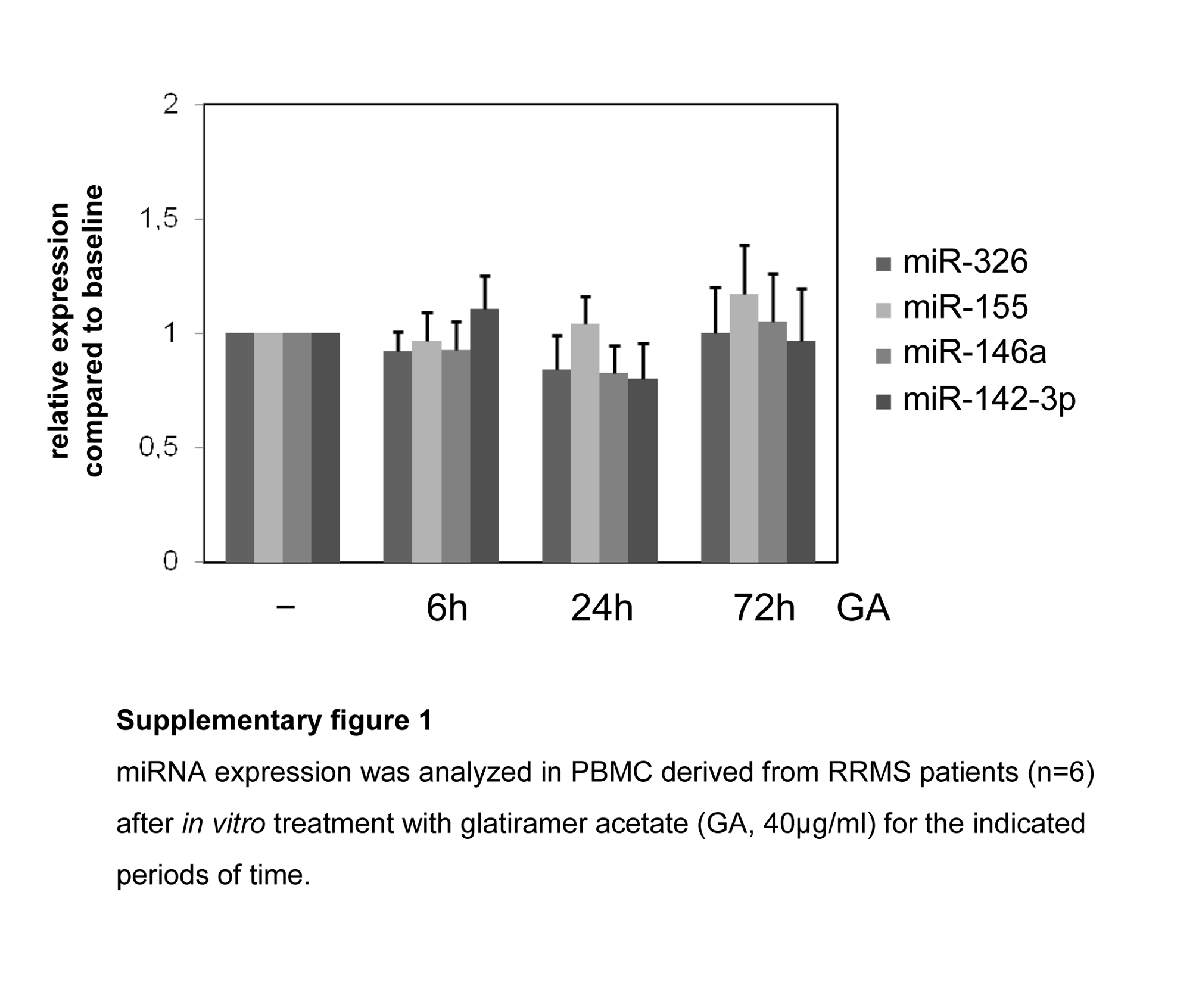

Supplement: Figure S1 — miRNA expression was analyzed in PBMC derived from RRMS patients (n = 6) after in vitro treatment with glatiramer acetate (GA, 40 µg/ml) for the indicated periods of time. (TIF) [file pone.0024604.s001.tif]
